# Malarial Fevers—Therapeutics

**Published:** 1884-09

**Authors:** J. T. Kent

**Affiliations:** St. Louis


### MALARIAL FEVERS—THERAPEUTICS.

PROF. J. T. KENT, A.M., M.D., ST. LOUIS.

By "malarial fevers" I mean such as are mixed, and not distinctively intermittent, generally denominated typho-malarial; exclusive of the variety which has, as a class, a clear apyrexia, such as are especially met in this city. This paper is intended to apply to the class of mixed fevers confined to St. Louis, to the cases blending from the complicated intermittent to the complicated typhoid. It is known that some of them take on a predominance of typhoid symptoms, and some of them a predominance of symptoms found in complicated intermittents. It is this hybrid state of things that causes us so much vexation. I have undertaken the task of furnishing the best guide to remedies for our own circumscribed work. I have not mentioned many remedies generally thought of great importance, because I have not found the symptoms indicating them. Should I go into remedies so seldom indicated, this paper might extend beyond endurance. Hence the remedies are those most useful.

*Antimonium crudum.*—The gastric derangement, nausea, and vomiting, great exhaustion, white tongue, and thirstlessness, constipation, or diarrhœa, must guide to this remedy. The concomitants, few or many, can seldom do away with indications for this remedy.

*Arnica.*—This is a frequently used remedy. The sore, bruised feeling all over the body; the patient complains of the "hard bed" and the aching, sore feeling in the whole body; the soreness compels him to move and he turns upon the other side, which in turn becomes sore and bruised, and compels him again to move; there is thirst and moaning; he cries for relief "or he will die!" There is great exhaustion and pain in the stomach and bowels, pressing and cutting pains in the stomach with nausea and vomiting; very often eructations, tasting like spoiled eggs, with bad taste in the mouth; diarrhœa of a blackish water, with bits of bloody, mucous stool; repugnance to food, milk, broth, and meat; coldness in the stomach, and, if there is a chill, it is preceded by great thirst.

*Arsenicum alb.*—Prostration, anxiety, and fear of death; extreme exhaustion, with thirst for water, little and often, for cold water, which causes nausea and vomiting; diarrhœa, stools scanty, dark, watery, offensive, with tenesmus, and the patient is covered with a cold sweat and blue spots. The tongue is dry and cracked, and the mouth and throat are parched and he wants only water enough to moisten the dry, mucous surfaces. In the beginning he goes from bed to bed, and is not relieved by the motion (unlike Rhus), yet his anxiety and restlessness compel him to move. The after midnight aggravation of fever and anxiety are especially guiding. The relief from warmth in general and warm drinks is also important. The burning in the stomach, bowels, mucous membranes, and skin, so common in many cases, is happily met by Arsenicum.

The involuntary stools generally point to Ars., but Arn. and Phos. have sometimes been indicated. The latter I have not often found indicated; occasionally the following symptoms have been present, indicating Phos.: The dry, burning mouth and tongue, with constant thirst for large quantities of ice-cold water, which is vomited when becoming warm in the stomach, or gurgling from the stomach down through the abdomen, causing an involuntary stool from a relaxed anus; hot head, desire to be magnetized, with overpowering fears; thinks he will see something coming from the corner of the room; bleeding from the nose, and septic exudation about the teeth (sordes); the face is blue, bloated, and Hippocratic; the terrible dryness is not relieved from drinking, and he wants a stream of cold water poured down his throat; there are stupor and delirium, and he slides down toward the foot of the bed (like Phos. ac. and Rhus). He answers no questions, or gives wrong answers to questions; great indifference.

*Baptisia.*—The peculiar sodden condition of the patient, with his besotted countenance, the face discolored and dusky, and the mental disquietude; his body he thinks is scattered over the bed, and he is trying to arrange the scattered members; he thinks his limbs are talking to each other; his answers are irregular, as if he were intoxicated; he seems to comprehend the question and makes an effort to answer, but falls to sleep, or into a stupor in the midst of the sentence; the tongue is foul and the mouth fetid; the delirium is greatest during the night; the functions are all sluggish, and the fever never runs very high; the pulse is often weak and compressible, sometimes the surface is cold.

In diphtheria the mucous membrane is dark and looks as if it might slough, and the exudation is dark; the surface is tumid and threatens to become gangrenous; finally dark, ragged, putrid ulcers form and the patient is too stupid to complain of pain; the tongue may be coated white or yellowish, white at first, but soon becomes dirty and brown and feels as if burnt or scalded and cracks; dark blood exudes. There is seldom much thirst, although if water be presented he will drink a large quantity and relapse into stupor. The typhoid abdomen and stools can be found under this remedy; yellow, mushy, and pasty, or bloody and very fetid; stools of pure blood or bloody mucus, exhausting and excoriating; involuntary stools. The tenderness and tympany of the abdomen are well marked. Baptisia is not a specific for typhoid fever, yet will cure promptly if given when the above symptoms are present. It is the remedy to begin as well as to finish the case. Arn., Hyos., Lach., Mur. ac., Opium, are especially related to it.

The *Arnica* patient forgets the word while speaking, but he does not begin his answer and fall into a profound sleep without finishing. Baptisia has the sore, bruised feeling of Arnica, but not the restlessness attending the soreness. The sensitiveness to pain is marked in Arnica and nearly lost in Baptisia. These remedies cannot be distinguished by the stools in many instances; both have dark, profuse, watery, fetid stools, and great soreness of the soft tissues as if bruised. The mental state and the besotted condition may be the only symptoms to base a choice upon.

A patient of mine was violently attacked with a chill; he moaned with pain and declared he would die; he purged almost involuntarily, a fetid, dark, watery stool; he would not answer me civilly, but said he was sore as if bruised. Between the violent abdominal pains he was stupid, as if drunk; when aroused he was snappish and his words did not express his probable intention.

The stool made me think of Baptisia, but Arn. has the same, also the mental state, hence it must be the most appropriate remedy. It broke his chill. The violence of the attack led me to anticipate a congestive chill, but the remedy quieted him very speedily.

Baptisia is often given, I find, where Hyoscyamus would be a more appropriate remedy. In the latter, the patient has a profound stupor, but when aroused he will answer correctly; the tongue is dry, black, and stiff, but there is not the tumid appearance of the mucous membranes as if sloughing would soon appear, or as if they would become gangrenous; Baptisia has involuntary stools, but not stools and urine like Hyoscyamus, nor does she attempt to expose the genitals in her delirium.

*Arsenicum* produces stools that cannot always be distinguished from those of Baptisia, but the thirst, so seldom in the latter, the extreme prostration and restlessness, will enable one to select the appropriate remedy. Arsenicum has the tendency to gangrene, but not the tumid, semi-transparent condition with the blueness. It has the bluish, or dusky aspect of the skin, but it is attended with a pinched condition of the countenance. Baptisia has a bluish, bloated condition of the face that is not so œdematous as that of Arsenicum. It is the result of venous stasis, not transudative, like that of the latter. Baptisia has not the heat of Arsenicum; both have involuntary stools, but Arsenicum has involuntary stools and urine; both have burning pain in the stomach, but Arsenicum has marked nausea, not found in Baptisia. Baptisia causes vomiting but without much nausea or effort. Baptisia seldom has much thirst, but when it is present, it is for a large quantity of cold water. It is not the important factor of the Arsenic thirst.

The *Arsenicum* delirium is a busy one; the *Baptisia* is passive. He will sometimes lie all day without moving if not disturbed; in the former, he is moving and is always in a hurry; the latter will do as advised, if he can; the former is irritable and wants his own way, and he is full of strange imaginations of vermin and burglars, and he has many fears.

*Hyoscyamus* corresponds to the most continued type in an advanced state; the tongue is dark or black, dry, and stiff; he is unable to put it out, the lips are dry and bleeding, the urine is passed in bed unconsciously, and there is much delirium. The patient answers questions correctly and lapses into stupor. (Arn. has the same, Baptisia goes into stupor in the midst of his attempted answer.) Hyoscyamus has cured my cases when the patient has passed into the state where it was impossible to arouse him. The profound stupor, pinched countenance, involuntary urine and stool, sliding down in the bed, picking at the bed-covers, picking the fingers, mark the case as a Hyoscyamus state when taken in connection with his having gone through the first symptoms mentioned.

*Muriatic acid* is one of the neglected remedies, yet one of the most valuable.

The *clinical* symptoms: *Clean, dry, red tongue,* sometimes *bluish,* is an important guiding symptom (not the slick, and shining tongue of Lach. and Kali bich.). There may be unconsciousness, moaning, and restlessness; thirst for acids and wine are also important; stools dark and mushy; urine passes involuntary; loud moaning, lower jaw dropped, tongue shrunken and dry like leather; hæmorrhage from the bowels. This remedy stands between Rhus and Bryonia. The patient is not made better from motion, like Rhus, and not made worse from motion, like Bryonia. It controls the septic processes and blood changes as well as Bry. or Rhus.

*Gelsemium.*—The heaviness of the limbs and thirstlessness; the bright eyes and contracted pupils; the active delirium; the extreme sinking feeling, paralytic weakness and fear of death; loquacity, talking in sleep. On the other hand, the face is pale and sallow and the pupils are dilated, yet the heaviness is always present. The mind symptoms and nervous prostration are most marked; the septic symptoms are not marked as in Ars., Bap., Arn., and Phos. The tongue trembles and is coated yellow. The many symptoms pointing to cerebral hyperæmia, point to *Gels.* and seldom to *Bell.* in these fevers. The sleeplessness is as prominent as any feature of these fevers, and *Gels.* is most generally its remedy. He is wide awake all night, "Not one wink of sleep last night," is the common answer (Op. Coff.). There is often pain running up the back, with contraction of dorsal muscles and stiffness, as if there was some meningeal complication; pain from spine to head and shoulders.

*Lycopus Virginiana.*—This remedy has been of great service to me. It is the remedy when the patient is stupid, will not answer questions, is waxy, cold, and has a pulse very low, yet full and large, soft and compressible; hæmorrhage from bowels, heavily loaded, tawny, expressionless face; if he has a fever it is not high, and he chokes and swallows; his eyes are expressionless; the veins are full and the face is bloated; the eyes seem to project from their sockets.

*Rhus tox.* is one of the most important remedies. The restlessness, *better from motion, great thirst*, dry tongue, sordes, reddish, watery, frothy stools in the morning, have been the symptoms calling for Rhus. The chilliness, like being dashed with cold water, and like cold water coursing through the veins, fever continues without sweat, and the restless aching, are often met. The patient often moves for relief; he finds a new place, and because he is completely exhausted he thinks he can rest; but soon the horrible aching and restlessness come on and he is compelled to move and find a new place, and this is continued night and day, and there is no rest and no sleep; there is a dry cough.

*Bryonia* is the remedy to be contrasted with Rhus. The pains may be severe, yet they are made worse by the slightest motion; he wants cold water in large quantity, but only occasionally; there is the dry, brown tongue, and the bowels are generally constipated; the stool is dry and hard as if burnt; the bowels are tympanitic and there is a foul, bitter taste in mouth; bleeding from the nose is common; there is often a dry cough and the right lung is often involved; there is delirium; he is busy and wants to be taken home; the fever and delirium are worse from nine o'clock till midnight. In Rhus the fever and delirium have been worse during the whole night and often continue all day. I see, by comparing my note-book, that several of my cases cured by *Rhus* had aggravation of mental and febrile symptoms at 5 A. M. and P. M. Bry. seldom has the twitching of muscles so common to Rhus, and of the two, urticaria, common in the beginning of some fevers, can only be found under the latter. The general aggravation from cold is characteristic of Rhus, but Bry. is oftener ameliorated by cold.

The long-lasting severe pain in the head I found, in my Bryonia cases, in the temples and eyes; improved by cold; the eyes were turgesced and the face was bloated and blue.

*Colchicum* was given in one case where the patient had an extreme disgust at the sight or smell of food, with marked benefit.

*Natrum sulphuricum* is a very important remedy. The patient says he has not been well for a long time; his sleep has not rested him, and his mouth has for a long time had a bad taste and his tongue is covered with a thick, yellow, pasty fur and tastes bitter. He now vomits bile and slime and has pain in the back of his head and his bones ache; the chill comes on and he runs into a quasi-continued fever, with chills occasionally; he has no appetite, his skin is yellow, and he has a yellow diarrhœa mixed with green slime.

*Ipecac.*—The aching in the back, thirstlessness, constant nausea, vomiting of green slime, red and pointed tongue, bitter taste; the case abused by quinine. Ipecac. is the remedy.

In the third and fourth week, some cases become very low; the tongue is sometimes red and slick, the papillæ all absorbed, and a smooth, slick, glossy surface on the tongue, and there is much vomiting of viscid, stringy mucus and bile. The patient is listless and delirious alternately.

*Eupatorium perf.* has improved cases when there was a bitter taste in the mouth, *aching in the bones as if they would break*, yellow skin, violent headache, day and night, worse during the scanty sweat, if there should be such a moisture; in many cases there is no perspiration, but great dryness of the skin and vomiting of bile.

When searching for remedies that correspond most faithfully to the fevers with absent sweating stage: Ars., Bapt., Bell., Bry., Cham., Colch., Eupator. perf., Gels., Hyos., Ign., Ipec., Kali bich., Lach., Lyc., Merc., Nitr. acid., Nux, Opium, Phos., Phos. acid., Rhus, and Sulph. may be consulted.

It will be found mostly that we are curing our patient from this list of remedies. When the exhaustion is the most marked feature, Arn., Ars., Bapt., China, Gels., Hyos., Lach., Lycopus, Phos., Phos. acid., and Rhus have been the most useful.

When the congested symptoms have been prominent, Arn. has been the remedy. It will be observed that I have not mentioned many of our so-called sheet anchors, as I have not found them of much service. Acon., Bell., and China have not been indicated in any of my cases. I have made use of bathing and inunction of lard in some protracted cases with great benefit, but never cathartics, stimulants, or quinine.

The single remedy is my reliance. I give the selected remedy every hour in these fevers, night and day, until improvement begins, and then I repeat cautiously.—*The Periscope.*

